# Use of nanoparticles, a modern means of drug delivery, against cryptosporidiosis

**DOI:** 10.5455/javar.2023.j726

**Published:** 2023-12-31

**Authors:** Faleh A. AlFaleh, Shameeran Salman Ismael, Liliana Aguilar-Marcelino, Fernando Edgar Martínez Silva, Tayyaba Ashraf, Rao Zahid Abbas, Warda Qamar

**Affiliations:** 1 Department of Biology, College of Science in Zulfi, Majmaah University, Majmaah, Saudi Arabia; 2 Medical Laboratory Sciences Department, College of Health Sciences, University of Duhok, Duhok, Iraq; 3 CENID-SALUD ANIMAL E INOCUIDAD, INIFAP, Morelos, México; 4 Investigador Pecuario, Campo Experimental Valles Centrales de Oaxaca CIRPAS-INIFAP, Oaxaca, Mexico; 5 Department of Parasitology, Faculty of Veterinary Science, University of Agriculture, Faisalabad, Pakistan

**Keywords:** *Cryptosporidium parvum*, nanoparticles, chitosan nanoparticles, silver nanoparticles, oocyst, viability

## Abstract

*Cryptosporidium* is a primary cause of waterborne epidemics, despite being previously considered only an opportunistic pathogen. The disease is associated with significant economic losses in humans and animals that are brought on by diarrhea, which frequently causes dehydration. Contact with diseased people or animals, as well as polluted water, is the major cause of infection. Different drugs are used to control the parasites. Nitazoxanide (NTZ), which is an anti-protozoan and anti-viral drug, can be used to control helminths, viruses, and protozoan parasites as a broad-spectrum antibiotic and has been approved by the food and drug authority (FDA). However, the problem is the development of resistance over a period of time in these parasites. Nanoparticles have received significant attention as possible anti-parasitic agents in recent years. By directing medications to specific cellular locations, targeted drug delivery minimizes the side effects of medications. Nanoparticles have demonstrated effectiveness against different *Cryptosporidium* species. Nanoparticles loaded with NTZ are found to be an effective remedy for *C. parvum* in young ones and decrease the oocyst count shed in the stools. Additionally, silver nanoparticles have proven to be effective against *C. parvum* by releasing silver ions that breach the cell wall of the oocyst, causing the escape of intracellular contents and the destruction of sporozoites within the oocyst. Implementing tiny particles for the purification of consuming water from *Cryptosporidium* is an economical and environmentally sustainable process. However, the use of nanoparticles in medicine requires more research.

## INTRODUCTION

*Cryptosporidium* parasites infect the digestive systems of many vertebrates. They are grouped into the phylum Apicomplexa. *Cryptosporidium* (*C*) causes diarrhea, and the disease is referred to as cryptosporidiosis [1–3]. With *Cryptosporidium*, 41 species and more than 60 genotypes are documented [4–6]. According to reports, *Cryptosporidium* is the second most common reason why newborns under the age of two get mild or serious diarrhea, behind rotavirus [7]. Cryptosporidiosis has public health significance because *Cryptosporidium* species are notorious for diarrhea caused by traveling [8,9] and are also accountable for diarrhea epidemics associated with water resources, e.g., waterparks, and municipal water supply [10,11]. In consumers, *C. hominis* and *C. parvum* are among the most common organisms, although *C. parvum* also infects ruminants [12]. The organisms *C. hominis* and *C. parvum* result in approximately a million human fatalities annually [13]. *C. parvum* is claimed to cause numerous financial losses due to its high mortality rates, lower efficiency, and costly medical care [14]. In developing countries, infection negatively affects malnourished children, retards their growth, causes hindrance in weight gain, and impairs physical development. The impact of cryptosporidiosis on animals can differ based on factors such as species, age, and overall health. In typical animals, the infection tends to be self-limiting and resolves within a week. However, in animals with weakened systems and pregnant animals, the infection may have more severe consequences, like weight loss, dehydration, and potentially even mortality [15].

This protozoon impacts not just individuals and wildlife but also a variety of birds. *C. meleagridis*, *C. baileyi*, and *C. galli* are pathogens that cause disease specifically in birds [16]. *C. meleagridis* and *C. galli* impact the digestive system and cause different levels of enteritis. *C. galli* infects and develops lesions in the proventriculus of poultry [17,18]*. C. baileyi shows different clinical forms of the disease: respiratory, enteritis (digestive), and renal. It develops lesions in various organs like the* gut, the kidneys, urinary tract, trachea, bronchi, air sacs, nasopharynx, conjunctiva, and bursa of Fabricius. Among birds, infection is frequently responsible for higher rates of death and illness [19,20] and has great economic importance. The life stages of *Cryptosporidium* are complicated and involve a single host for both sexual and asexual stages. Thick-walled oocysts are resistant to adverse environmental conditions and contaminate the water sources that are accidentally ingested by humans, animals, or birds [21,22]. The acidic environment of the intestine stimulates the excystation of oocysts, and sporozoites are released [23,24]. Sporozoites attach to the host’s cells of epithelial tissue and cause a parasitophorous vacuole to form. The parasite passes through different developmental forms like trophozoite, Type I meront, Type I merozoite, Type II meront, and Type II merozoite [1,^25^,26]. Type II merozoite forms microgamont (male) and macrogamont (female), which are round in shape and vary in size from 4 to 6 µm [27,28]. Microgametes fertilize the nearby macrogamont, which develops into a diploid zygote. Within the intestinal lumen, these zygotes develop into oocysts, which either re-infect the same host or may be excreted from the body through feces into the external environment.

Treatment of disease involves various drugs like nitazoxanide (NTZ), paromomycin, azithromycin, a combination of azithromycin and paromomycin, and rifaximin. NTZ is a US Food and Drug Administration (FDA)-approved drug whose active chemical is nitrothiazolyl-salicylamide [29]. The drug is an anti-protozoan/anti-viral agent that has wide-spectrum antibacterial activity against viruses, bacteria, helminths, and protozoan parasites [30–32]. It is effective in mild infections but does not give good results in moderate and heavy infections [33,34]. Paromomycin belongs to the group aminoglycosides, and it is found effective in children and adults to treat different levels of infection [35–37]. Paromomycin decreases the protozoa and oocysts shedding in stools and treats diarrhea. Though it does not eliminate the infections, symptoms appear again in half of the patients [38,39]. Azithromycin is an antibiotic that belongs to the macrolide group and has been found effective in some infections [40–42]. Rifaximin is an FDA-approved antibiotic that is used to treat travelers’ diarrhea [43–45]. Unfortunately, its action is restricted to people who are most at risk for serious illness. In recent years, researchers have initiated numerous initiatives to identify secure and effective parasite therapies. Here, we discuss many novel targeted mechanisms for treating cryptosporidiosis, along with current and emerging therapeutic approaches.

### Need for nanotechnology

In spite of primary research efforts, to this point, since most parasitic infections do not elicit a strong immune response, there is currently no vaccine that is successful against any of the majority of common parasitic illnesses. Subsequently, the use of anti-parasitic agents is a very important strategic tool for fighting parasitic infections [46,47]. However, researchers instituted anti-parasitic drugs more than 50 years ago. Furthermore, while some drugs are efficient, the majority of anti-parasitic medications fall well short of the modern definition of a “drug” when it comes to acceptability and treatment, the duration of the remedy, precision, and the consent of the patient [48]. In contradiction, the cost of the latest drug development and drug discovery against parasitic infections is extremely low in comparison to the many other fields of study. In actuality, just 1% of the 1223 novel medications that were introduced to the marketplace between 1975 and 1996 were created specifically for the treatment of tropical invading parasitic infections like trypanosomiasis, malaria, and leishmaniasis. Until 2000, researchers allocated just 0.1% of global health research funding to the search for anti-parasitic drugs, reflecting a lack of concern for infections caused by parasites. So, coming up with new ways to give currently available anti-parasitic drugs that make them more effective, more accurate, easier to tolerate, and able to treat a wider range of parasitic diseases is a great idea that might help stop the disease pandemic [49,50]. Given the commonness of parasitic diseases and the bad side effects that come with current anti-parasitic drugs, it is important to look into new drugs that are highly effective, don’t cause side effects, and are not too expensive. Certain limitations afflict conventional preparations such as suspension or emulsion. There may be a need for a few innovative providers who can perfectly meet the requirements of a drug-transporting device due to factors like excessive dosage and low accessibility, first bypass impact, intolerance, instability, and variations in plasma drug degrees. Currently, nanoparticle delivery machines have been proposed as colloidal drug carriers. Nanoparticles may show size-related characteristics that vary extensively from the ones discovered in materials found in bulk and fine particles [51]. The key properties of nanoparticles are: (1) increased bioavailability by improving aqueous solubility; (2) increasing ½-lives for elimination from the body; (3) increasing specificity for their specific receptors; and (4) placing the drug wherever it acts within the human body. This results in a gradual decrease in the amount of drug needed and the toxicity of drugs, permitting the secure delivery of toxic drugs and the protection of surrounding tissues and cells from being damaged [52]. Therefore, in this review, we discussed various nanoparticles that were used against *C. parvum* parasites.

### Nanoparticles

With time, nanoparticles have drawn great attention. Nanoparticles are the basic component of nanotechnology, and their dimensions vary between 1 and 100 nanometers (nm). Scientists synthesized nanoparticles from organic material, metals like carbon, and metal oxides [53]. Nanoparticles are available in different dimensions, shapes, and sizes in addition to their material [54]. They can be single-dimensional, like graphene, two-dimensional in nature, like carbon nanotubes (CNT), three-dimensional, like gold nanoparticles, or zero-dimensional, like nanodots. Their shapes range from spherical to cylindrical, tubular, conical, hollow core, spiral, and flat. They can also be crystalline, with homogeneous, regular surfaces, or amorphous, with uneven surfaces [55]. Nanotechnology has a vast range of applications in medicine, cosmetics, crockery, electronic appliances, and the aerospace industry.

### Types of nanoparticles

There are different kinds of particles organic, inorganic, and metal-based.

#### Organic nanoparticles

The polymers of ferritin, liposomes, dendrimers, and micelles are known as organic nanoparticles. These particular nanoparticles are biodegradable, stable, non-toxic, and capable of transporting drugs. The hollow centers of liposomes and micelles, referred to as nanocapsules, are radiation, light, and heat sensitive [56,57]. These special properties make them a good medium for the delivery of drugs. These types of nanoparticles are commonly used in medical fields because they are efficient. In targeted drug delivery systems, nanoparticles are administered to specific organs of the body. The properties of nanoparticles depend upon the composition of the material, shape, size, flexibility of the material, and surface quality [58]. Nanoparticles are composed of various materials, i.e., lipids, and a vast range of synthetic polymers of various compounds. The most popular artificial polymers that are utilized to create nanoparticles are poly (lactic-co-glycolic) acid (PLGA), dextrans, and polyanhydrides, while natural polymers include elastin-like polypeptides. The use of these nanoparticles depends on the method of preparation, their toxicity, and their compatibility with loaded drugs [59]. Nanoparticles are modified by binding with ligands to their surface, e.g., peptides, antibodies, aptamers, and then applied to specific tissues like cancer cells [60,61]. The efficiency of nanoparticles also depends on their shape and structure. The efficiency of rod-shaped nanoparticles on targeted tissues is higher than that of spherical nanoparticles [62,63].

#### Inorganic nanoparticles

There are no carbon atoms in nanoparticles that are inorganic. They are further categorized into metal-based and metal oxide-based nanoparticles. Metal-based nanoparticles are prepared by converting the metals into nanoparticles either through constructive or destructive methods. All kinds of metals can be used for the formation of nanoparticles [64]. Nanoparticles are synthesized commonly from gold (Au), silver (Ag), zinc (Zn), lead (Pb), copper (Cu), cobalt (Co), cadmium (Cd), and aluminum (Al). These nanoparticles have specific characteristics like high surface charge, pore size, surface area-to-volume ratio, and surface charge density. Characteristics of different nanoparticles vary; e.g., aluminum nanoparticles show sensitivity to heat, sunlight, and moisture, have a large surface area, and are unstable [65]. Furthermore, silver nanoparticles exhibit lower reactivity and can serve as effective disinfectants and antimicrobial agents [66]. Gold nanoparticles are highly reactive and unstable [67]. Copper nanoparticles (CuNPs) are flammable solids, conductors of heat and electricity, and ductile [68]. Zinc nanoparticles are resistant to corrosion, can be employed in treating bacterial and fungal infections, and provide protection against ultraviolet radiation [69]. Cobalt nanoparticles are highly unstable, toxic, and can absorb magnetic waves and microwaves [70]. Metal oxide nanoparticles are formed by the modification of their corresponding metal-based nanoparticles. At room temperature, nanoparticles of iron (Fe) oxidize to iron oxide (Fe_2_O_3_) in the presence of oxygen. Metal oxide nanoparticles are formed when there is an increase in their reactivity and efficiency [71]. Aluminum oxide (Al_2_O_3_), cerium oxide (CeO_2_), iron oxide (Fe_2_O_3_), magnetite (Fe_3_O_4_), silicon dioxide (SiO_2_), titanium oxide (TiO_2_), and zinc oxide (ZnO) [72–75]. In contrast to the corresponding metals, these small particles have unique characteristics.

#### Carbon-based nanoparticles

Carbon atoms are an integral part of carbon-based nanoparticles. Graphene, fullerenes, nanotubes of carbon (CNT), carbon black, carbon nanofibers, and carbon that are activated are the different types of carbon nanomaterials. Fullerenes (C_60_) are composed of carbon atoms. Fullerene molecules are round in form. There are about 28–1,500 atoms of carbon aggregated with each other by sp^2^ hybridization to form fullerenes. The diameters of the single-layered and multi-layered fullerenes are 8.2 nm and 4–36 nm, respectively [76]. It is a carbon allotrope. The diameter of a graphene sheet is approximately 1 nm. It is a two-dimensional, hexagon-shaped honeycomb structure network composed of carbon atoms [77]. CNTs are formed by winding a graphene nanofoil with a honeycomb-like shape made of carbon atoms into elongated cylinders. Scientists form CNTs by winding the graphene nanofoil into elongated cylinders. A single-layer CNT has a diameter of 0.7 nm, while multi-layered CNTs have a diameter of 100 nm. CNTs range in dimension from various millimeters (mm) to a few micrometers (µm). Half-fullerene molecules may seal both ends of a CNT. This material—nanofoils wound into an oval or cup-shaped substitute for standard cylindrical tubes—makes up carbon nanofiber. Nanotubes of carbon are comparable to these. Made of carbon, carbon black is a kind of amorphous substance. The size of it ranges from 20 to 70 nm, giving it an elliptical form. Because of the particles’ strong reactions and interactions, carbon black particles were created [78].

### Applications

Numerous uses have developed for nanoparticles but they are mostly desirable in medicine and drug delivery systems.

### Medicine

Nanotechnology is a recent advancement in the medical field. Nanoparticles deliver drugs specifically to body cells [79]. We can minimize the side effects of drugs by placing them in a specific area. This method decreases the amount of drug, cost, and side effects. Nanotechnology helps in tissue engineering and reproduction. Nanotechnology can be used to replicate broken tissue and reconstruct it, a process known as the tissue engineering method. Tissue engineering has replaced the traditional methods of treatment, e.g., artificial implants and organ transplants. The development of bone-based CNT scaffolds is a particularly notable example [80]. Nanoparticles speed up the healing process, so they can be used as antiseptics, e.g., silver nanoparticles. Silver nanoparticles are also used to control bacteria and fungi and act as an antibacterial agent. Silver nanoparticles are highly effective against bacteria and fungi, which are resistant to drugs, and their sensitivity varies according to each species [81]. These nanoparticles show high efficacy against MRSA (methicillin-resistant *Staphylococcus aureus*), *Streptococcus pyogenes,* and methicillin-resistant *Staphylococcus epidermidis*. Silver nanoparticles break down the cell walls of these bacteria, e.g., *Klebsiella pneumonia* and *Salmonella typhi*. So, they are less effective against them [82]. A combination of chitosan (CS)-Ag nanoparticles has high potency to fight against *Escherichia coli* and is less effective against *Candida albicans,* which is due to variations in the structural composition of the outer wall of the cell and the presence of different functional groups around the cell wall of various types of bacteria [83]. Ag NPs work by attaching themselves to cells, breaking down enzymes and nucleic acids, and producing harmful free radicals and reactive oxygen species (ROS) that cause oxidative stress [84–87]. They function by inhibiting the synthesis of biofilms and the destruction of bacteria in previously formed biofilms [88]. The combination of Ag NPs and Quercetin has a synergistic effect and is also safe to use as an antibacterial against *E. coli* and *S. aureus* [89].

### Drug delivery system

**Figure 1.**
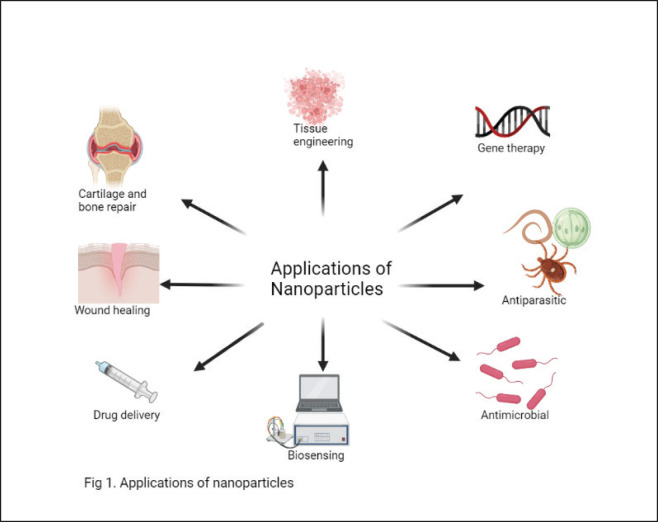
Application of nanoparticle against *Cryptosporidium*.

Nanoparticles are utilized for the delivery of different medicines to target sites. The antibacterial effect of drugs can be enhanced by their loading and conjugation with the nanoparticles. *Naegleria fowleri,* the causative organism of meningoencephalitis, can be cured by using Amphotericin B, Fluconazole, and Nystatin loaded on Ag NPs [90]. The increase in the efficacy of drugs is due to an increase in their total amount and their availability in the targeted tissue. The toxicity of Ag NPs can be reduced by the slow release of silver ions in the host cells [91]. Ag NPs loaded with oseltamivir are highly effective against influenza virus strain H1N1 because the antiviral activity of oseltamivir is enhanced due to the generation of ROS [92]. Silver nanoparticles can also be used to control Chagas disease, which results in extensive necrosis caused by *Trypanosoma cruzi* prepared from the reducing agent Xylan [93]. Drugs conjugated with silver nanoparticles show more efficiency than drugs used alone and are also less harmful and toxic to host tissues [94]. Silver nanoparticles are synthesized using plant hexane extracts from *Phaselous coccinus*. Ag NPs inhibit the penetration of host cells by a virus that enhances their antiviral character against COxB4, HAV-1, and HAV-10 [95]. Figure 1 illustrates the applications of nanotechnology in different fields, i.e*.,* gene therapy, tissue engineering, drug delivery, biosensing, anti-parasitic antimicrobials, wound healing, cartilage repair, and bone repair.

**Figure 2.**
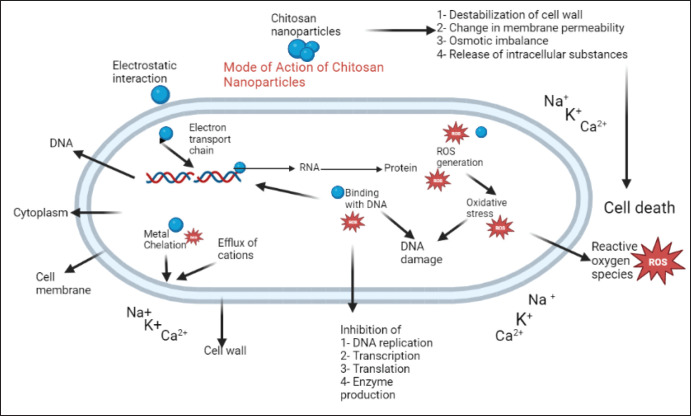
Mode of action of chitosan nanoparticles against *Cryptosporidium* oocyst (created by bio render).

Scientists have been using nanoparticles for various purposes in recent years, particularly as anti-parasitic agents [96–99]. In medicine, the extensive use of Chitosan Nanoparticles (CS NPs) is due to their compatibility with the environment and their bacteriostatic characteristics [100,101]. CS particles are prepared from N-acetyl-d-glucosamine and d-glucosamine subunits. Chemically, CS NPs are polysaccharides that are formed by the deacetylation of chitin in a basic environment. They have no adverse effects, can be used as antitumor, antibacterial, and antifungal agents, are involved in wound healing, and act as immune system stimulants [102,103]*.* In nanomedicine, CS NPs are most preferably employed for the loading of drugs due to their specific characteristics for carrying drugs, which also increase the availability of drugs at target sites and the duration of their action. They can solubilize in an aqueous medium and produce positively charged ions. This property allows for their most common and efficient use with greater success [104]. It also increases the permeability of molecules through various surfaces, like mucosal surfaces [105]. The oocysts of *Cryptosporidium* are resistant to the environment, have high stability, and continue to thrive for a longer duration of time, up to 12 months, which is why they produce most waterborne diseases [106]. The oocyst wall of *Cryptosporidium* is very resistant and hard, and it requires a longer duration of time for the action of CS NPs for their complete elimination. There is a positive charge on the surface of CS NPs and a negative charge on the oocyst wall of *Cryptosporidium,* which is why they stick to each other firmly, and this phenomenon increases the duration of action of nanoparticles, which is highly toxic for *Cryptosporidium* oocysts. They interact with nucleic acids, disrupt their helical structure, and also produce ROS that impose oxidative stress and lead to oocyst inactivation [107,108]. Figure 2 explains the mode of action of CS NPs.

NTZ causes a remarkable decrease in the shedding of oocysts in immunosuppressed and immunocompetent mice. On the 11th day, the percentage decrease in oocyst passing in immunocompetent and immunosuppressed groups is 42.01% and 32.42%, respectively, and on the 19th day, the reduction percentage is 57.1% and 41.06%, respectively [30]. CS NPs loaded with NTZ are a very effective remedy for *C. parvum* in young ones and decrease the number of parasites shedding in the environment [109]. Conjugation of CS nanosuspension with Bupravaquone will increase the duration of the drug in the intestine and its availability, which is why this combination is more effective as compared to Bupravaquone alone [110,111]. The mucoadhesive property of CS NPs is responsible for enhancing the duration and improved action of drugs in the digestive tract and decreasing their excretion from the gut. In the gastrointestinal tract, CS NPs attach to the intestinal wall and attack the pathogen directly [112]. Praziquantel loaded on CS NPs is a very effective treatment for larval and mature stages of *Schistosoma* with a half-dose rate [113].

*C. parvum* continues to shed oocysts for 30 days in Swiss albino mice [114]. It is also reported that oocysts can be shed for up to 3 weeks in this study [115]. When severe infection occurs with *Cryptosporidium,* watery diarrhea is tenacious and lasts for five weeks [116]. It is reported that CS NPs along with *Nigella sativa* (black cumin) reduce the shedding of oocysts and limit their spread both in immunocompetent and immunosuppressed mice on the 27th day after infection, with a reduction percentage of 79.16% and 73.33%, respectively [111,117]. CS NPs reduce the shedding of oocysts at 18 days after infection in immunocompetent and immunosuppressed mice by 17.3% and 11.7%, respectively [111]. *N. sativa* has various therapeutic effects like antioxidant, neuroprotective, immunopotentiation, antiasthmatic, antitumor, anti-inflammatory, and antimicrobial [118]. It is revealed that the seeds of *N. sativa* act as phytotherapeutic agents against plasmodium and also have antioxidant properties [119]. *N. sativa* extracts, in combination with honey, can treat cutaneous leishmaniasis (CL) better than honey alone [120]. A combination of *N. sativa* with CS NPs decreases the number of parasites by 77.5% [113]. CS NPs result in a reduction of parasite counts in the brain, spleen, and liver by 6.42%, 17.66%, and 23.94%, respectively, in mice infected with the *Toxoplasma* RH strain [121]. CS NPs combined with polyvinyl alcohol are used to break the link between *Cryptosporidium* sporozoites and the cells of the intestine *in vitro* and CS NPs show high efficiency against *Cryptosporidium* [122]. CS NPs conjugated with NTZ decrease the number of deaths in immunosuppressed mice compared to the untreated control group [121].

Therapeutic effects of various drugs are determined by evaluating the different histopathological improvements in the liver, intestine, and lungs in intestinal and extra-intestinal forms of *Cryptosporidium* infection. In cryptosporidiosis, there is shortening and then destruction of the villi of the small intestine, intensive inflammation, ulcers on the mucosal surface, and complete loss of the brush border, so absorption of nutrients does not occur. Dysplastic changes in the intestine were also observed [114,^123^,124]. Specific toxins produced by pathogens adversely affect the epithelial cells and lead to the atrophy of villi and complete loss of brush borders [125]. NTZ alone can be used for the treatment, but it shows a mild improvement in the pathological condition of the intestine [126]. However, the combination of CS NPs with NTZ causes significant improvement in the pathological changes by rejoining the atrophied intestinal villi and also improving the liver picture. Immunocompetent mice show better results as compared to immunosuppressed mice [124]. Table 1 illustrates the efficacy of different nanoparticles against *Cryptosporidium parvum* oocysts.

### Use of silver nanoparticles (Ag NPs)

**Figure 3.**
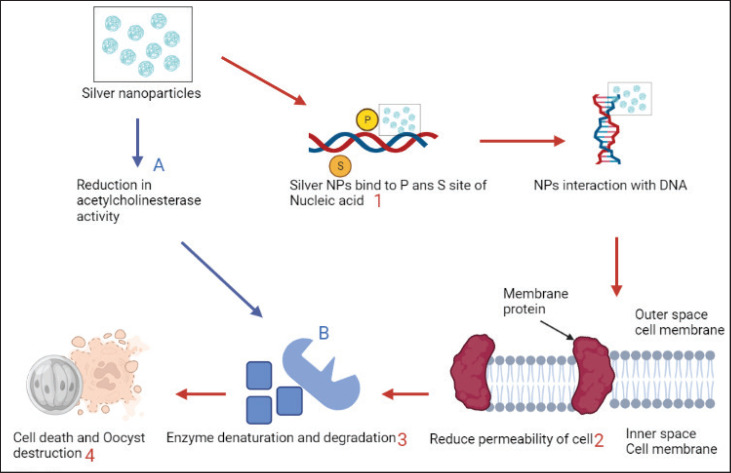
Mode of action of silver nanoparticles against *C. parvum* oocyst. (Created by Biorender.) Description: Ag NPs bind to P and S site of DNA and Proteins (1) reduce the permeability of cell membrane (2), Enzyme degradation (3), which leads to cell death (4). On the other hand, Ag NPs reduce acetylcholinesterase activity (A) that leads to enzyme denaturation and ultimately cell death (B).

Silver nanoparticles show high efficacy in fighting against various pathogens and advancements in the field of science. Ag NPs are used in medicine and are capable of controlling bacteria, fungi, and protozoa [139–141]. The mechanism of action of Ag NPs to fight against bacteria and parasites involves the formation of silver ions (Ag+), which results in the production of ROS and leads to oxidative stress [142]. Moreover, nanoparticles are small, which provides a greater surface area and a longer duration of contact for binding to the bacteria. It leads to the slow release of silver ions and then triggers ROS [143]. Ag NPs act on *C. parvum* by releasing silver ions, which enter the oocyst by breaking the cell wall and causing the expulsion of all intracellular contents and the destruction of sporozoites within the oocyst. The use of nanoparticles for the purification of drinking water is a cost-effective and environmentally friendly procedure [127,^144^,145]. Ag NPs kill bacteria by exerting their antibacterial effect through cytotoxic and cell-inhibitory action [146]. Ag NPs are also used to control various protozoa like *Leishmania*, *Giardia*, *Entamoeba*, *Toxoplasma*, *Plasmodium,* and insect larvae and helminths [112,147–149]. Ag NPs decrease the spread of *Leishmania* parasites by blocking their metabolic action and the destruction of promastigotes [150]. There are various mechanisms by which AgNPs cause the destruction of the oocysts of *Cryptosporidium* species. Glycoprotein and lipophosphoglycan, responsible for virulence, are destroyed by the formation of ROS and consequently due to oxidative stress. This leads to the oocysts becoming inactive and may not cause parasitic infection [151]. The size of nanoparticles is very small, so they can readily move throughout the cellular membrane, lead to adverse effects on parasites, and result in their killing [152]. Ag NPs can cause toxic effects by binding with the molecules of DNA and destroy the double-helical structure by disrupting the cross-linkage of DNA strands, as shown in Figure 3 [153]. Nanoparticles also interrupt normal biochemical reactions [154]. Silver nanoparticles can decrease the number of oocysts and their duration of survival in the environment, which reduces their propagation to new hosts.

**Table 1.** Efficacy of different nanoparticles against *Cryptosporidium parvum* oocysts.

Nanoparticle	Country	Test used	Sample extracted	Concentration/dose rate	Results/efficacy	Reference
Silver nanoparticles	United Kingdom	Counting of oocyst, sporozoites, and empty shells by PCM.	Fecal sample	500 µg/ml	Reduction in oocyst count 83.3% ± 3% to 33.3% ± 17.5%.	[127]
Chitosan nanoparticles loaded with nitazoxanide	Egypt	Oocyst count	Fecal sample	200 mg/ml CS NPs loaded with NTZ	The reduction percentage in oocyst is 75.7%.	[128]
Chitosan nanoparticles	Egypt	Oocyst shedding by using Modified Ziehl-Neelson stain	Fecal sample	500 µg/ml 1500 µg/ml 3000 µg/ml 5000 µg/ml 7000 µg/ml	Oocyst destruction rate is 68.88% 86% 91% 97.3% 99.87% at different dose rate mentioned in previous column after 72 h of exposure.	[122]
Gold nanoparticles	Thailand	Oocyst count by HPF.	Stool sample	1cc	Oocysts reduce from 8.8 ± 1.2 oocysts/HPF to 4.7 ± 1.2 oocysts/HPF.	[129]
Silver nanoparticles	Egypt	Oocyst count	Water sample	0.1 ppm	Reduction percentage in oocyst count is 87% at 30 min of exposure.	[130]
Chitosan nanoparticles	China	Oocyst count	Fecal sample	1 mg/Kg/day	Reduction percentage in oocyst count is observed 75.54% (4275.90 ± 703 oocyst)	[131]
Silver nanoparticles	Egypt	Oocyst count	Fecal sample	5 mg/Kg/day	Oocyst reduced from 13733 ± 3885 at 14 days post-infection to 247 ± 94 at 28-day post infection.	[132]
Gold nanoparticles	Canada	Oocyst count	Fecal sample	25 µL	Significant reduction in oocyst count.	[133]
Silver nitrate nanoparticles	America	Oocyst count	Stool sample	100 mg/L	The mean no. of oocysts per 10 mg stool is 10^1.9^. Statically significant reduction in oocyst count. Modification of excystation behavior is 90%.	[134]
CP2-NP-906 Poly lactic-co-glycolic acid (PLGA) loaded with compound thymidylate synthase-dihydrofolate reductase (TS-DHFR) conjugated with *Cryptosporidium* specific proteins (CP2).	USA	Cell culture to estimate the anti-parasitic effect on sporozoites and intracellular forms.	*Cryptosporidium* infected cells.	Size of CP2-NP-906 is 100 to 300 nM for delivery of drug.	Reduction in the level of parasites is by 200-fold in cell culture.	[135]
Silver nanoparticles	Egypt	Oocyst count by SEM.	Fecal sample	0.54–1mg.	LC50 for 3 h of exposure is 0.54–1 mg.	[136]
Copper nanoparticles (CuO)	Egypt	Oocyst count by SEM.	Fecal sample	0.72mg.	LC50 for 3 h of exposure is 0.72 mg.	[136]
Indinavir loaded modified nanoparticles (Ab-TMR-IND-Np). Anti-*Cryptosporidium* IgG polyclonal antibody conjugated with tetramethylrhodamine labelled nanoparticle (D,L-lactide-co-gycolide).	Italy	Histogram of human cell line is examined by IFA.	HCT-8 cells (Human ileocecaladenocarcinoma tumor cell line).	50 µM Ab-TMR-IND-Np to the HCT-8 cells at the time of oocyst addition. 50 µM Ab-TMR-IND-Np added to cryptosporidium infected cells.	Complete inhibition of oocyst excystation and no infection occur. The reduction percentage of intracellular parasites depends upon the time of exposure i.e. 25%–30% after 24 h, 51% after 48 h, 67% after 72 h, and 70% after 96 h.	[137]
Chitosan NAG nanoparticles (N-acetyl-d-glucosamine units) and chitosan Mix	USA	IFA	Human ileocecal adenocarcinoma cells (HCT-8 cell line) and human colonic adenocarcinoma cells (Caco-2 cell line).	500 µg/ml of chitosan NAG and Chitosan mix.	Chitosan NAG reduces level of parasites in HCT-8 61.2% and Caco-2 44.1%. Chitosan mix reduces intracellular forms in HCT-8 78.8% and Caco-2 67.9%. Paromomycin sulphate reduces the level of intracellular parasites in HCT-8 44.7% and Caco-2 32.9%.	[138]

Abbreviations: Scanning electron microscope (SEM), High Power Field (HPF), Immuno fluorescent Assay (IFA), Phase contrast microscopy (PCM), HCT-8 cells (Human ileocecaladenocarcinoma tumor cell line).

Table 2 describes the activity of oocysts of *C. parvum* exposed to the silver nanoparticles and the control group, which are not treated with Ag NPs for different durations of exposure. Mortality and activity of oocysts exposed to various concentrations of Ag NPs for different intervals of time and in the control group (*p* < 0.05) show significant variation. If the Ag NPs are exposed to oocysts for 1 to 4 h at a dose rate of 0.05 ppm, they show better results than if exposed for 30 min. The percentage reduction in oocyst activity at different exposure times (30 min at 1 ppm and 2 h at 0.05 ppm) is 97.3% and 78.3%, respectively [130]. This shows significant reduction percentages, which are given in the table.

Different concentrations of Ag NPs can be used to limit the viability of oocysts, as the maximum reduction percentage can be observed at a dose rate of 1 ppm and the minimum reduction percentage is reported at 0.05 ppm. It is described that a wide range of doses are used to render the oocysts inactive, from 0.005 to 500 µg/ml, in a dose-dependent manner [127]. Exposure to Ag NPs reduces their feasibility in feces and their further propagation [155]. Nanoparticles also disrupt the structure of the oocyst’s cell wall, rendering them inactive. It is reported that low-dose Ag NPs with smaller sizes show more stimulatory effects than large-size nanoparticles. Ag NPs are available most commonly in the range of 8.2 to 42.1 nm. The toxic effects and availability of nanoparticles for the purification of water are changed by the presence of fecal material, organic contaminants, and heavy metals [127,156]. Higher concentrations of chlorides combine with the Ag NPs and form insoluble aggregates, which decrease the exposure time of the pathogen to the nanoparticles and reduce its antiparasitic effects against *C. parvum* [157,158].

**Table 2.** Exposure of Ag NPs for different duration of time and reduction percentage in *Cryptosporidium* viability.

Ag NPs Dose rate	Duration of exposure	Mortality (%)	Viability of *Cryptosporidium* oocyst	
			Exposed sample	Control
1.0 ppm	4 h	82.2	5.1 ± 0.5^e^	28.3 ± 2.9^a^
	2 h	82.3	5.6 ± 0.5^de^	31.6 ± 2.8^b^
	1 h	90.9	2.6 ± 0.7^d^	28.6 ± 3.0^a^
	30 min	97.2	0.8 ± 0.4^c^	28.6 ± 3.0^ab^
0.1 ppm	4 h	93.3	1.9 ± 0.2^cd^	28.3 ± 2.9^a^
	2 h	92.7	2.3 ± 0.3^d^	31.6 ± 2.8^b^
	1 h	94.4	1.6 ± 0.1^c^	28.6 ± 3.0^a^
	30 min	93.3	1.9 ± 0.8^cd^	28.6 ± 3.0^ab^
0.05 ppm	4 h	90.1	2.8 ± 0.6^d^	28.3 ± 2.9^a^
	2 h	78.3	4.0 ± 0.8^cd^	31.6 ± 2.8^b^
	1 h	89.9	2.9 ± 0.5^d^	28.6 ± 3.0^a^
	30 min	79.0	6.0 ± 1.4^c^	28.6 ± 3.0^ab^

Readings having no common superscript show significant variation (*p* < 0.05) [130].

## Use of copper and gold nanoparticle

CuNPs have anti-microbial activity against various types of bacteria, e.g., *Salmonella enteric*, *S. aureus*, *Campylobacter jejuni*, *E. coli*, *Listeria monocytogenes, Aspergillus niger*, etc. [159–161]. The copper oxide nanoparticles can be employed for the control of parasites with an inhibitory concentration (IC_50_) of 0.13 mg/l for *Entamoeba histolytica* and 0.72 mg/l for *C. parvum* [155]. It is also reported that exposure to copper oxide nanoparticles for 180 min at a concentration of 0.6 mg/ml can render 97% of *Giardia lamblia* parasites inactive [162]. The mode of action of CuNPs involves their interaction with sulfhydryl groups, which then leads to protein denaturation, which ultimately results in the death of pathogens [160]. CuNPs decrease the permeability of the cell membrane, production of ROS, disruption of DNA molecules, protein denaturation, and lipid peroxidation, and hence prove toxic for bacterial pathogens [163]. Drugs kill microbes by using the programmed cell death phenomenon, also known as apoptosis, and use different types of caspase enzymes for killing [164]. Exposure to CuNPs at different concentrations leads to the stimulation of cell death by activation of caspase-3 activity in the protoscoleces of *Echinococcus granulosus*. CuNPs exposure for 48 h can inactivate the protoscoleces by inducing regulation of caspase enzymes of 20.5%, 32.3%, and 36.1% at dose rates of 250, 500, and 750 mg/ml, respectively [165].

The effectiveness of gold nanoparticle solutions as anti-infective agents is extensively elaborated. The utility of the improvement of novel antibacterial drugs is stated [166]. Furthermore, the expertise of its hobby in opposition to pathogenic protozoa is restricted. Thus, the writers conduct an initial examination to evaluate the impact of a gold nanoparticle solution on *Cryptosporidium* oocyst. The authors use this version to evaluate the impact of gold nanoparticles on cells, as in the previously posted papers. Briefly, the writer used the 30 fecal samples with *Cryptosporidium* oocyst for trial. Every fecal sample was divided into two parts by naive manipulation and combined with 1 cc of gold nanoparticles [167,168]. The authors evaluate the reduction in the number of *Cryptosporidium* oocysts in each group. At the start, the average number of *Cryptosporidium* oocysts per high-power field (HPF) is 8.8 ± 1.2 oocysts/HPF. The reduction of the implied *Cryptosporidium* oocyst quantity after checking may be discovered. The common change after manipulation in the naïve and gold nanoparticle solution combined groups is 1.2 ± 0.5 oocysts/HPF and 4.7 ± 1.2 oocysts/HPF, respectively. There is a huge distinction in the reduction of the number of oocysts between these two groups (*P* < 0.05; *t*-check). This preliminary report would possibly suggest that a gold nanoparticle solution has an impact on the inactivation of *Cryptosporidium* oocyst. This will verify the idea that attaching gold nanoparticles to indinavir may enhance its *in vitro* efficiency against *C. parvum* [137].

## Future perspective

*Cryptosporidium* is a parasite made up of protozoan cells that can seriously harm both humans and animals’ gastrointestinal systems. It is highly resistant to traditional water treatment methods and can survive in aquatic environments for extended periods, posing a significant public health threat [169]. Nanoparticles have demonstrated their potential as a tool in the fight against *Cryptosporidium* because of their distinct chemical and physical characteristics. Nanoparticles are able to enter the parasite’s cell membrane, disrupting its integrity and inhibiting its growth and reproduction [170]. One potential future perspective for the use of nanoparticles against *Cryptosporidium* is the development of nanoparticle-based water filtration systems. These systems could be used to remove *Cryptosporidium* and other pathogens from drinking water, improving public health outcomes [171]. Another potential application of nanoparticles is in the process of evolving targeted drug delivery systems for the treatment of *Cryptosporidium* infections. By encapsulating drugs within nanoparticles, it may be possible to lessen potential side effects and increase medication effectiveness, as well as increase drug stability and shelf life [172]. Overall, the use of nanoparticles against *Cryptosporidium* holds great promise for improving water quality and treating *Cryptosporidium* infections. However, further study is required to completely comprehend the safety and efficacy of these approaches and to develop practical and cost-effective solutions for their implementation [173].

## Conclusion

In the past few years, it has been observed that nanoparticles, particularly CS-based and silver nanoparticles, are capable of delivering drugs to the target sites with more efficiency and have fewer side effects and toxicity. In this review based upon previous literature, it is revealed that various medicines embedded in CS and Ag NPs are highly efficient anticryptosporidial agents employed to control *Cryptosporidium* species due to an increase in their availability and longer duration of action at target sites. Ag NPs and CS NPs are effective alternatives to drugs and safer to use. Nanotechnologies help us find effective disinfectants that are safe to use and also less costly for the purification of water by killing *C. parvum* and various other parasites and bacteria. However, nanoparticles are costly, and more research is required to find their practical uses in clinics and to find out the dose rate and safe concentrations of CS NPs and Ag NPs for a specific duration of time to fight against this parasite.

## List of abbreviations

*C. Cryptosporidium*; FDA, Food and Drug Authority; PLGA, Poly Lactic-co-Glycolic Acid; Au, Gold; Ag, Silver; Zn, Zinc; Pb, Lead; Cu, Copper; Co, Cobalt; Cd, Cadmium; Al, Aluminum; Al_2_O_3_, Aluminum oxide; CeO_2_, Cerium oxide; Fe_2_O_3_, Iron oxide; Fe_3_O_4_, Magnetite; SiO_2_, Silicon dioxide; TiO_2_, Titanium oxide; Zno, Zinc oxide; CNT, Carbon Nanotubes; mm, Millimeters; µm, Micrometers; nm, Nanometers; MRSA, Methicillin-Resistant *Staphylococcus aureus*; ROS, Reactive Oxygen Species; *E. coli*, *Escherichia coli*; CS NPs, Chitosan Nanoparticles; NTZ, Nitazoxanide; Ag NPs, Silver Nanoparticles; CL, Cutaneous Leishmaniasis; ppm, Parts per million; CuNPs, Copper nanoparticles; IC_50_, Inhibitory Concentration; Au Nps, Gold Nanoparticle; HPF, High Power Field; SEM, Scanning Electron Microscope; IFA, Immuno Fluorescent Assay; PCM, Phase Contrast Microscopy; HCT-8 cells, Human ileocecaladenocarcinoma tumor cell line; TS-DHFR, Thymidylate Synthase-Dihydrofolate Reductase; Ab-TMR-IND-Np, Indinavir loaded modified nanoparticles.

## References

[1] Fayer R. (2010). Taxonomy and species delimitation in Cryptosporidium. Exper Parasitol.

[2] Chen X, Saeed NM, Ding J, Dong H, Kulyar MF-E-A, Bhutta ZA (2022). Molecular epidemiological investigation of cryptosporidium sp., Giardia duodenalis, Enterocytozoon bieneusi and Blastocystis sp. Infection in Free-ranged Yaks and Tibetan Pigs on the Plateau. Pak Vet J.

[3] Xuehan L, Lei D, Zhong Z, Ziyao Z, Xuefeng Y, Zhixing A. (2021). Identification of a New MLST subtype of cryptosporidium muris from a brown rat (Rattus norvegicus) in China. Kafkas Üniv Vetr Fakült Derg.

[4] Qi M, Wang R, Ning C, Li X, Zhang L, Jian F (2011). Cryptosporidium spp. in pet birds: genetic diversity and potential public health significance. Exper Parasitol.

[5] Liu X, He T, Zhong Z, Zhang H, Wang R, Dong H (2013). A new genotype of cryptosporidium from giant panda (Ailuropoda melanoleuca) in China. Parasitol Int.

[6] Kváč M, Hofmannová L, Hlásková L, Květoňová D, Vítovec J, McEvoy J (2014). *Cryptosporidium erinacei* n. sp.(Apicomplexa: Cryptosporidiidae) in hedgehogs. Vet Parasitol.

[7] Kotloff KL, Blackwelder WC, Nasrin D, Nataro JP, Farag TH, van Eijk A (2012). The global enteric multicenter study (GEMS) of diarrheal disease in infants and young children in developing countries: epidemiologic and clinical methods of the case/control study. Clin Infect Dis.

[8] Nair P, Mohamed JA, DuPont HL, Figueroa JF, Carlin LG, Jiang Z-D (2008). Epidemiology of cryptosporidiosis in North American travelers to Mexico. Am J Trop Med Hyg.

[9] Patel AR, Oheb D, Zaslow TL. (2018). Gastrointestinal prophylaxis in sports medicine. Sports Health.

[10] Yoder JS, Craun GF, Calderon RL. (2008). Surveillance for waterborne disease and outbreaks associated with recreational water use and other aquatic facility-associated health events---United States, 2005–2006. Centers for Disease Control and Prevention (CDC).

[11] Hlavsa MC, Roberts VA, Kahler AM, Hilborn ED, Wade TJ, Backer LC (2014). Recreational water–associated disease outbreaks—United States, 2009–2010. Morb Mort Weekly Rep.

[12] Keleş İ, Ekinci G, Tüfekçi E, Çitil M, Güneş V, Aslan Ö (2022). Etiological and predisposing factors in calves with Neonatal Diarrhea: a clinical study in 270 case series [1] Neonatal İshalli Buzağılarda Etiyolojik ve Predispoze Faktörler: 270 Olgu Serisinde Klinik Bir Çalışma. Kafkas Univ Vet Fakult Derg.

[13] Dokainish H, Teo K, Zhu J, Roy A, AlHabib KF, ElSayed A (2017). Global mortality variations in patients with heart failure: results from the International Congestive Heart Failure (INTER-CHF) prospective cohort study. Lancet Global Health.

[14] Shahiduzzaman M, Daugschies A. (2012). Therapy and prevention of cryptosporidiosis in animals. Vet Parasitol.

[15] Kim S, Yu D-H, Jung S, Kang J, Park K, Chae J-B (2021). Biological factors associated with infectious diarrhea in calves. Pak Vet J.

[16] Ryan U, Xiao L, Read C, Zhou L, Lal AA, Pavlasek I. (2003). Identification of novel Cryptosporidium genotypes from the Czech Republic. Appl Environ Microbiol.

[17] Gharagozlou MJ, Dezfoulian O, Rahbari S, Bokaie S, Jahanzad I, Razavi ANE. (2006). Intestinal cryptosporidiosis in turkeys in Iran. J Vet Med Series A.

[18] Holubová N, Sak B, Horčičková M, Hlásková L, Květoňová D, Menchaca S (2016). Cryptosporidium avium n. sp.(Apicomplexa: Cryptosporidiidae) in birds. Parasitol Res.

[19] Santos M, Peiró JR, Meireles MV. (2005). Cryptosporidium infection in ostriches (Struthio camelus) in Brazil: clinical, morphological and molecular studies. Braz J Poult Sci.

[20] Pagès-Manté A, Pages-Bosch M, Majó-Masferrer N, Gómez-Couso H, Ares-Mazás E. (2007). An outbreak of disease associated with cryptosporidia on a red-legged partridge (Alectoris rufa) game farm. Avian Pathol.

[21] El-Khodery SA, Osman SA. (2008). Cryptosporidiosis in buffalo calves (Bubalus bubalis): prevalence and potential risk factors. Trop Anim Health Prod.

[22] Campbell I, Tzipori AS, Hutchison G, Angus KW. (1982). Effect of disinfectants on survival of cryptosporidium oocysts. Vet Record.

[23] Widmer G, Klein P, Bonilla R. (2007). Adaptation of Cryptosporidium oocysts to different excystation conditions. Parasitology.

[24] Matos LV, McEvoy J, Tzipori S, Bresciani KD, Widmer G. (2019). The transcriptome of Cryptosporidium oocysts and intracellular stages. Sci Rep.

[25] Smith HV, Nichols RAB, Grimason AM. (2005). *Cryptosporidium excystation* and invasion: getting to the guts of the matter. Trends Parasitol.

[26] Borowski H, Thompson RCA, Armstrong T, Clode PL. (2010). Morphological characterization of *Cryptosporidium parvum* life-cycle stages in an in vitro model system. Parasitology.

[27] Yang S, Healey MC, Du C, Zhang J. (1996). Complete development of *Cryptosporidium parvum* in bovine fallopian tube epithelial cells. Infect Immun.

[28] Chen F, Huang K-H. (2009). *In vitro* cultivation model of *Cryptosporidium parvum* in MDCK cells and its development. Chinese J Parasitol Parasit Dis.

[29] Checkley W, White AC, Jaganath D, Arrowood MJ, Chalmers RM, Chen X-M (2015). A review of the global burden, novel diagnostics, therapeutics, and vaccine targets for cryptosporidium. Lancet Infect Dis.

[30] Amadi B, Mwiya M, Musuku J, Watuka A, Sianongo S, Ayoub A (2002). Effect of nitazoxanide on morbidity and mortality in Zambian children with cryptosporidiosis: a randomised controlled trial. Lancet.

[31] Rossignol JF, Kabil SM, El–Gohary Y, Younis AM. (2006). Effect of nitazoxanide in diarrhea and enteritis caused by Cryptosporidium species. Clin Gastroenterol Hepatol.

[32] McLeod C, Morris PS, Snelling TL, Carapetis JR, Bowen AC. (2014). Nitazoxanide for the treatment of infectious diarrhoea in the Northern Territory, Australia 2007–2012. Rural Remote Health.

[33] Doumbo O, Rossignol JF, Pichard E, Traore HA, Dembele M, Diakite M (1997). Nitazoxanide in the treatment of cryptosporidial diarrhea and other intestinal parasitic infections associated with acquired immunodeficiency syndrome in tropical Africa. Am J Trop Med Hyg.

[34] Diptyanusa A, Sari IP. (2021). Treatment of human intestinal cryptosporidiosis: a review of published clinical trials. Int J Parasitol Drugs Drug Resist.

[35] Hashmey R, Smith NH, Cron S, Graviss EA, Chappell CL (1997). Cryptosporidiosis in Houston, Texas: a report of 95 cases. Med Baltimore.

[36] Stockdale HD, Spencer JA, Blagburn BL. (2007). Prophylaxis and chemotherapy. *Cryptosporidium* and cryptosporidiosised.

[37] Helmy YA, Hafez HM. (2022). Cryptosporidiosis: from prevention to treatment, a narrative review. Microorganisms.

[38] Clinton White Jr A, Chappell CL, Sikander Hayat C, Kimball KT, Flanigan TP, Goodgame RW. (1994). Paromomycin for cryptosporidiosis in AIDS: a prospective, double-blind trial. J Infect Dis.

[39] Masur H, Brooks JT, Benson CA, Holmes KK, Pau AK, Kaplan JE. (2014). Prevention and treatment of opportunistic infections in HIV-infected adults and adolescents: updated Guidelines from the Centers for Disease Control and Prevention, National Institutes of Health, and HIV Medicine Association of the Infectious Diseases Society of America. Clin Infect Dis.

[40] Hicks P, Zwiener RJ, Squires J, Savell V. (1996). Azithromycin therapy for *Cryptosporidium parvum* infection in four children infected with human immunodeficiency virus. J Pediat.

[41] Kadappu KK, Nagaraja MV, Rao PV, Shastry BA. (2002). Azithromycin as treatment for cryptosporidiosis in human immunodeficiency virus disease. J Postgrad Med.

[42] Pantenburg B, Cabada MM, White Jr AC. (2009). Treatment of cryptosporidiosis. Exp Rev Anti-Infect Ther.

[43] Amenta M, Nogare ERD, Colomba C, Prestileo TS, Lorenzo FD, Fundaro S (1999). Intestinal protozoa in HIV-infected patients: effect of rifaximin in *Cryptosporidium parvum* and Blastocystis hominis infections. J Chemother.

[44] Gathe Jr JC, Mayberry C, Clemmons J, Nemecek J. (2008). Resolution of severe cryptosporidial diarrhea with rifaximin in patients with AIDS. JAIDS J Acquir Immun Defic Syndr.

[45] Khan SM, Witola WH. (2023). Past, current, and potential treatments for cryptosporidiosis in humans and farm animals: a comprehensive review. Front Cell Infect Microbiol.

[46] Datta AK, Datta R, Sen B. (2008). Antiparasitic chemotherapy. Drug targets in kinetoplastid parasites. Advances in Experimental Medicine and Biologyed.

[47] Sueth-Santiago V, Decote-Ricardo D, Morrot A, Freire-de-Lima CG, Lima MEF. (2017). Challenges in the chemotherapy of Chagas disease: looking for possibilities related to the differences and similarities between the parasite and host. World J Biol Chem.

[48] Watkins BM. (2003). Drugs for the control of parasitic diseases: current status and development. Trends Parasitol.

[49] Pink R, Hudson A, Mouriès M-A, Bendig M. (2005). Opportunities and challenges in antiparasitic drug discovery. Nat Rev Drug Discov.

[50] Kandeel M, Akhtar T, Zaheer T, Ahmad S, Ashraf U, Omar M. (2022). Anti-parasitic applications of nanoparticles: a review. Pak Vet J.

[51] Buzea C, Pacheco II, Robbie K. (2007). Nanomaterials and nanoparticles: sources and toxicity. Biointerphases.

[52] Irving B. (2007). Nanoparticle drug delivery systems. Innov Pharm Biotechnol.

[53] Hasan S. (2277). A review on nanoparticles: their synthesis and types. Res J Recent Sci 2015.

[54] Cho EJ, Holback H, Liu KC, Abouelmagd SA, Park J, Yeo Y. (2013). Nanoparticle characterization: state of the art, challenges, and emerging technologies. Mol Pharm.

[55] Machado S, Pacheco JG, Nouws HPA, Albergaria JT, Delerue-Matos C. (2015). Characterization of green zero-valent iron nanoparticles produced with tree leaf extracts. Sci Total Environ 2015.

[56] Tiwari DK, Behari J, Sen P. (2008). Application of nanoparticles in waste water treatment. World Appl Sci.

[57] Gehrke I, Geiser A, Somborn-Schulz A. (2015). Innovations in nanotechnology for water treatment. Nanotechnol Sci Applicat.

[58] Muthu MS, Rawat MK, Mishra A, Singh S. (2009). PLGA nanoparticle formulations of risperidone: preparation and neuropharmacological evaluation. Nanomed Nanotechnol Biol Med.

[59] MacEwan SR, Chilkoti A. (2012). Digital switching of local arginine density in a genetically encoded self-assembled polypeptide nanoparticle controls cellular uptake. Nano Lett.

[60] Farokhzad OC, Cheng J, Teply BA, Sherifi I, Jon S, Kantoff PW (2006). Targeted nanoparticle-aptamer bioconjugates for cancer chemotherapy in vivo. Proc Nat Acad Sci.

[61] El-Dawy K, Mohamed D, Abdou Z. (2022). Nanoformulations of pentacyclic triterpenoids: chemoprevention and anticancer. Int J Vet Sci.

[62] Barua S, Yoo J-W, Kolhar P, Wakankar A, Gokarn YR, Mitragotri S. (2013). Particle shape enhances specificity of antibody-displaying nanoparticles. Proc Nat Acad Sci.

[63] Cao J, Choi J-S, Oshi MA, Lee J, Hasan N, Kim J (2019). Development of PLGA micro-and nanorods with high capacity of surface ligand conjugation for enhanced targeted delivery. Asian J Pharm Sci.

[64] Salavati-Niasari M, Davar F, Mir N. (2008). Synthesis and characterization of metallic copper nanoparticles via thermal decomposition. Polyhedron.

[65] Geetha P, Latha MS, Pillai SS, Deepa B, Kumar KS, Koshy M. (2016). Green synthesis and characterization of alginate nanoparticles and its role as a biosorbent for Cr (VI) ions. J Mol Struct.

[66] Hulteen JC, Treichel DA, Smith MT, Duval ML, Jensen TR, Van Duyne RP. (1999). Nanosphere lithography: size-tunable silver nanoparticle and surface cluster arrays. J Phys Chem.

[67] Syed B, Prasad NMN, Satish S. (2016). Endogenic mediated synthesis of gold nanoparticles bearing bactericidal activity. J Microsc Ultrast.

[68] Ryu C-H, Joo S-J, Kim H-S (2016). Two-step flash light sintering of copper nanoparticle ink to remove substrate warping. Appl Surf Sci.

[69] Bogutska KI, Sklyarov YP, Prylutskyy Y. (2013). Zinc and zinc nanoparticles: biological role and application in biomedicine. Ukrain Bioorg Acta.

[70] Bau VM, Bo X, Guo L. (2017). Nitrogen-doped cobalt nanoparticles/nitrogen-doped plate-like ordered mesoporous carbons composites as noble-metal free electrocatalysts for oxygen reduction reaction. J Energy Chem.

[71] Tai CY, Tai C-T, Chang M-H, Liu H-S. (2007). Synthesis of magnesium hydroxide and oxide nanoparticles using a spinning disk reactor. Indust Eng Chem Res.

[72] Munuswamy DB, Madhavan VR, Mohan M. (2015). Synthesis and surface area determination of alumina nanoparticles by chemical combustion method. Int J ChemTech Res.

[73] Bajpai SK, Jadaun M, Tiwari S. (2016). Synthesis, characterization and antimicrobial applications of zinc oxide nanoparticles loaded gum acacia/poly (SA) hydrogels. Carbohyd Polym.

[74] Kaynar ÜH, Şabikoğlu I, Kaynar SÇ, Eral M. (2016). Modeling of thorium (IV) ions adsorption onto a novel adsorbent material silicon dioxide nano-balls using response surface methodology. Appl Rad Isotop.

[75] Samy A, Hassan H, Elsherif H. (2022). Effect of nano zinc oxide and traditional zinc (oxide and sulphate) sources on performance, bone characteristics and physiological parameters of broiler chicks. Int J Vet Sci.

[76] Tenne R. (2002). Fullerene-like materials and nanotubes from inorganic compounds with a layered (2-D) structure. Colloids Surf Physicochem Eng Aspect.

[77] Huang X, Boey F, Zhang HUA. (2010). A brief review on graphene-nanoparticle composites. Cosmos.

[78] Fawole OG, Cai XM, MacKenzie AR. (2016). Gas flaring and resultant air pollution: a review focusing on black carbon. Environl Pollut.

[79] Ganesh K, Archana D, Preeti K. (2013). Review article on targeted polymeric nanoparticles: an overview. Am J Adv Drug Deliv.

[80] Mudshinge SR, Deore AB, Patil S, Bhalgat CM. (2011). Nanoparticles: emerging carriers for drug delivery. Saudi Pharm J.

[81] Roy A, Bulut O, Some S, Mandal AK, Yilmaz MD (2019). Green synthesis of silver nanoparticles: biomolecule-nanoparticle organizations targeting antimicrobial activity. RSC Adv.

[82] Nanda A, Saravanan M. (2009). Biosynthesis of silver nanoparticles from *Staphylococcus aureus* and its antimicrobial activity against MRSA and MRSE. Nanomed Nanotechnol Biol Med.

[83] Biao L, Tan S, Wang Y, Guo X, Fu Y, Xu F, Zu Y, Liu Z. (2017). Synthesis, characterization and antibacterial study on the chitosan-functionalized Ag nanoparticles. Mater Sci Eng.

[84] Malarkodi C, Rajeshkumar S, Paulkumar K, Jobitha GG, Vanaja M, Annadurai G. Biosynthesis of semiconductor nanoparticles by using sulfur reducing bacteria Serratia nematodiphila. Adv Nano Res.

[85] Zhao R, Lv M, Li Y, Sun M, Kong W, Wang L (2017). Stable nanocomposite based on PEGylated and silver nanoparticles loaded graphene oxide for long-term antibacterial activity. ACS Appl Mater Interf.

[86] Kędziora A, Speruda M, Krzyżewska E, Rybka J, Łukowiak A, Bugla-Płoskońska G. (2018). Similarities and differences between silver ions and silver in nanoforms as antibacterial agents. Int J Mol Sci.

[87] Aziz S, Abdullah S, Anwar H, Latif F, Mustfa W. (2021). Effect of engineered Nickel Oxide nanoparticles on antioxidant enzymes in freshwater fish, Labeo rohita. Pak Vet J.

[88] Mohanta YK, Biswas K, Jena SK, Hashem A, Abd_Allah EF, Mohanta TK. (2020). Anti-biofilm and antibacterial activities of silver nanoparticles synthesized by the reducing activity of phytoconstituents present in the Indian medicinal plants. Front Microbiol.

[89] Sun D, Zhang W, Mou Z, Chen Y, Guo F, Yang E (2017). Transcriptome analysis reveals silver nanoparticle-decorated quercetin antibacterial molecular mechanism. Acs Appl Mate Interf.

[90] Rajendran K, Anwar A, Khan NA, Siddiqui R. (2017). Brain-eating amoebae: Silver nanoparticle conjugation enhanced efficacy of anti-amoebic drugs against *Naegleria fowleri*. ACS Chem Neurosci.

[91] Shin JU, Gwon J, Lee S-Y, Yoo HS. (2018). Silver-incorporated nanocellulose fibers for antibacterial hydrogels. ACS Omega.

[92] Li Y, Lin Z, Zhao M, Xu T, Wang C, Hua L (2016). Silver nanoparticle based codelivery of oseltamivir to inhibit the activity of the H1N1 influenza virus through ROS-mediated signaling pathways. ACS Appl Materials Interf.

[93] Brito TK, Silva Viana RL, Gonçalves Moreno CJ, da Silva Barbosa J, Lopes de Sousa Júnior F, Campos de Medeiros MJ (2020). Synthesis of silver nanoparticle employing corn cob xylan as a reducing agent with anti-*Trypanosoma cruzi* activity. Int J Nanomed.

[94] Anwar A, Siddiqui R, Hussain MA, Ahmed D, Shah MR, Khan NA. (2018). Silver nanoparticle conjugation affects antiacanthamoebic activities of amphotericin B, nystatin, and fluconazole. Parasitol Res.

[95] Haggag EG, Elshamy AM, Rabeh MA, Gabr NM, Salem M, Youssif KA (2019). Antiviral potential of green synthesized silver nanoparticles of Lampranthus coccineus and Malephora lutea. Int J Nanomed.

[96] Khan I, Khan M, Umar MN, Oh DH. (2015). Nanobiotechnology and its applications in drug delivery system: a review. IET Nanobiotechnol.

[97] Khan I, Zaneb H, Masood S, Ashraf S, Rehman HF, Rehman HU (2022). Supplemental selenium nanoparticles-loaded to chitosan improves meat quality, pectoral muscle histology, tibia bone morphometry and tissue mineral retention in broilers. Pak Vet J.

[98] Aziz S, Abdullah S, Anwar H, Latif F. (2022). DNA damage and oxidative stress in economically important fish, bighead carp (*Hypophthalmichthys nobilis*) exposed to engineered copper oxide nanoparticles. Pak Vet J.

[99] Zarnab S, Javed MT, Gul A-HST, Mahmood MS. (2022). The chicken in-house environment can be improved by the use of nanotechnology. Pak Vet J.

[100] Kong M, Chen XG, Xing K, Park HJ. (2010). Antimicrobial properties of chitosan and mode of action: a state of the art review. Int J Food Microbiol.

[101] Katas H, Raja MAG, Lam KL. (2013). Development of chitosan nanoparticles as a stable drug delivery system for protein/siRNA. Int J Biomat.

[102] Goy RC, Britto Dd, Assis OBG. (2009). A review of the antimicrobial activity of chitosan. Polímeros.

[103] De Marchi JGB, Jornada DS, Silva FK, Freitas AL, Fuentefria AM, Pohlmann AR (2017). Triclosan resistance reversion by encapsulation in chitosan-coated-nanocapsule containing α-bisabolol as core: development of wound dressing. Int J Nanomed.

[104] Akakuru OU, Louis H, Amos PI, Akakuru OC, Nosike EI, Ogulewe EF. (2018). The chemistry of chitin and chitosan justifying their nanomedical utilities. Biochem Pharmacol.

[105] Luppi B, Bigucci F, Cerchiara T, Zecchi V. (2010). Chitosan-based hydrogels for nasal drug delivery: from inserts to nanoparticles. Expert Opin Drug Deliv.

[106] Omarova A, Tussupova K, Berndtsson R, Kalishev M, Sharapatova K. (2018). Protozoan parasites in drinking water: A system approach for improved water, sanitation and hygiene in developing countries. Int J Environ Res Public Health.

[107] Searcy KE, Packman AI, Atwill ER, Harter T. (2006). Capture and retention of *Cryptosporidium parvum* oocysts by Pseudomonas aeruginosa biofilms. Appl Environ Microbiol.

[108] Mohammed MA, Syeda JTM, Wasan KM, Wasan EK. (2017). An overview of chitosan nanoparticles and its application in non-parenteral drug delivery. Pharmaceutics.

[109] Sedighi F, Abbasali Pourkabir R, Maghsood AH, Fallah M. (2016). Comparison of therapeutic effect of anti-Cryptosporidium nano-nitazoxanide (NTZ) with free form of this drug in neonatal rat. Avicenna J Clin Med.

[110] Kayser O. (2001). A new approach for targeting to *Cryptosporidium parvum* using mucoadhesive nanosuspensions: research and applications. Int J Pharm.

[111] Mohamed WA, Koura EA, Rabee I, Hammam OA, Ismail HM. (2019). The efficacy of chitosan nanoparticle alone versus conjugated with Nigella sativa (EL Baraka seed oil) against *Cryptosporidium parvum* in infected immunocompetent and immunosuppressed mice. WJ Pharm Pharm Sci.

[112] Said DE, Elsamad LM, Gohar YM. (2012). Validity of silver, chitosan, and curcumin nanoparticles as anti-Giardia agents. Parasitol Res.

[113] Elawamy WE, Mohram AF, Naguib MM, Ali HS, Kishik SM, Hendawi FF. (2019). Therapeutic role of chitosan nanoparticles in murine schistosomiasis mansoni. J Med Plants Res.

[114] Abdou AG, Harba NM, Afifi AF, Elnaidany NF. (2013). Assessment of *Cryptosporidium parvum* infection in immunocompetent and immunocompromised mice and its role in triggering intestinal dysplasia. Int J Infect Dis.

[115] Lacroix S, Mancassola R, Naciri M, Laurent F. (2001). *Cryptosporidium parvum-*specific mucosal immune response in C57BL/6 neonatal and gamma interferon-deficient mice: role of tumor necrosis factor alpha in protection. Infect Immun.

[116] Borad A, Ward H. (2010). Human immune responses in cryptosporidiosis. Future Microbiol.

[117] O’Hara SP, Chen X-M. (2011). The cell biology of Cryptosporidium infection. Microbes Infect.

[118] Agarwal R, Kharya MD, Shrivastava R. (1979). Antimicrobial and anthelmintic activities of the essential oil of Nigella sativa Linn. Indian J Exper Biol.

[119] Okeola VO, Adaramoye OA, Nneji CM, Falade CO, Farombi EO, Ademowo OG. (2011). Antimalarial and antioxidant activities of methanolic extract of Nigella sativa seeds (black cumin) in mice infected with Plasmodium yoelli nigeriensis. Parasitol Res.

[120] Nilforoushzadeh MA, Hejazi SH, Zarkoob H, Shirani-Bidabadi L, Jaffary F. (2010). Efficacy of adding topical honey-based hydroalcoholic extract Nigella sativa 60% compared to honey alone in patients with cutaneous leishmaniasis receiving intralesional glucantime. J Skin Leishman.

[121] Etewa SE, El-Maaty DAA, Hamza RS, Metwaly AS, Sarhan MH, Abdel-Rahman SA (2018). Assessment of spiramycin-loaded chitosan nanoparticles treatment on acute and chronic toxoplasmosis in mice. J Parasitic Dis.

[122] Ahmed SA, El-Mahallawy HS, Karanis P. (2019). Inhibitory activity of chitosan nanoparticles against *Cryptosporidium parvum* oocysts. Parasitol Res.

[123] Abu El Ezz NMT, Khalil FAM, Shaapan RM. (2011). Therapeutic effect of onion (Allium cepa) and cinnamon (Cinnamomum zeylanicum) oils on cryptosporidiosis in experimentally infected mice. Global Vet.

[124] Abdelhamed EF, Fawzy EM, Ahmed SM, Zalat RS, Rashed HE. (2019). Effect of nitazoxanide, artesunate loaded polymeric nano fiber and their combination on experimental cryptosporidiosis. Iran J Parasitol.

[125] Soufy H, Nadia M, Nasr SM, Abd El-Aziz TH, Khalil FAM, Ahmed YF (2017). Effect of Egyptian propolis on cryptosporidiosis in immunosuppressed rats with special emphasis on oocysts shedding, leukogram, protein profile and ileum histopathology. Asian Pac J Trop Med.

[126] Sadek G, El-Aswad B. (2014). Role of COX-2 in pathogenesis of intestinal cryptosporidiosis and effect of some drugs on treatment of infection. Res J Parasitol.

[127] Cameron P, Gaiser BK, Bhandari B, Bartley PM, Katzer F, Bridle H. (2016). Silver nanoparticles decrease the viability of *Cryptosporidium parvum* oocysts. Appl Environ Microbiol.

[128] Moawad HSF, Hegab MHAE-H, Badawey MSR, Ashoush SE, Ibrahim SM, Ali AAE-LS. (2021). Assessment of chitosan nanoparticles in improving the efficacy of nitazoxanide on cryptosporidiosis in immunosuppressed and immunocompetent murine models. J Parasit Dis.

[129] Joob B, Wiwanitkit V. (2014). Effect of gold nanoparticle solution on cryptosporidium oocyst: the world first report. Ann Trop Med Public Health.

[130] Hassan D, Farghali M, Eldeek H, Gaber M, Elossily N, Ismail T. (2019). Antiprotozoal activity of silver nanoparticles against *Cryptosporidium parvum* oocysts: New insights on their feasibility as a water disinfectant. J Microbiol Methods.

[131] Rahman SU, Gong H, Mi R, Huang Y, Han X, Chen Z. (2022). Chitosan protects immunosuppressed mice against *Cryptosporidium parvum* infection through TLR4/STAT1 signaling pathways and gut microbiota modulation. Front Immunol.

[132] Hassan ZR, Salama DEA, Ibrahim HF. (2022). Apoptotic changes in the intestinal epithelium of Cryptosporidium-infected mice after silver nanoparticles treatment versus nitazoxanide. J Parasit Dis.

[133] Jain S, Huang Z, Dixon BR, Sattar S, Liu J. (2019). *Cryptosporidium parvum* oocyst directed assembly of gold nanoparticles and graphene oxide. Front Chem Sci Eng.

[134] Abebe LS, Su Y-H, Guerrant RL, Swami NS, Smith JA. (2015). Point-of-use removal of *cryptosporidium parvum* from water: independent effects of disinfection by silver nanoparticles and silver ions and by physical filtration in ceramic porous media. Environ Sci Technol.

[135] Mukerjee A, Iyidogan P, Castellanos-Gonzalez A, Cisneros JA, Czyzyk D, Ranjan AP (2015). A nanotherapy strategy significantly enhances anticryptosporidial activity of an inhibitor of bifunctional thymidylate synthase-dihydrofolate reductase from Cryptosporidium. Bioorg Med Chem Lett.

[136] Abdel Halim AS, Amr BM. (2015). Antiparasitic activity of silver and copper oxide nanoparticles against Entamoeba Histolytica and *Cryptosporidium Parvum* cysts. J Egypt Soc Parasitol.

[137] Bondioli L, Ludovisi A, Tosi G, Ruozi B, Forni F, Pozio E (2011). The loading of labelled antibody-engineered nanoparticles with Indinavir increases its in vitro efficacy against *Cryptosporidium parvum*. Parasitology.

[138] Mammeri M, Chevillot A, Thomas M, Polack B, Julien C, Marden JP (2018). Efficacy of chitosan, a natural polysaccharide, against *Cryptosporidium parvum in vitro* and *in vivo* in neonatal mice. Exper Parasitol.

[139] Prabhu S, Poulose EK. (2012). Silver nanoparticles: mechanism of antimicrobial action, synthesis, medical applications, and toxicity effects. Int Nano Lett.

[140] Dosoky R, Kotb S, Farghali M. (2015). Efficiency of silver nanoparticles against bacterial contaminants isolated from surface and ground water in Egypt. J Adv Vet Anim Res.

[141] Kailasa SK, Park T-J, Rohit JV, Koduru JR. (2019). Antimicrobial activity of silver nanoparticles. Nanoparticles in pharmacotherapyed.

[142] Fauss EK, MacCuspie RI, Oyanedel-Craver V, Smith JA, Swami NS. (2014). Disinfection action of electrostatic versus steric-stabilized silver nanoparticles on *E. coli* under different water chemistries. Colloids Surf B Biointerfaces.

[143] Rai M, Yadav A, Gade A. (2009). Silver nanoparticles as a new generation of antimicrobials. Biotechnol Adv.

[144] Brame J, Li Q, Alvarez PJJ. (2011). Nanotechnology-enabled water treatment and reuse: emerging opportunities and challenges for developing countries. Trends Food Sci Technol.

[145] Qu X, Brame J, Li Q, Alvarez PJJ. (2013). Nanotechnology for a safe and sustainable water supply: enabling integrated water treatment and reuse. Accounts Chem Res.

[146] Zheng J, Wu X, Wang M, Ran D, Xu W, Yang J. (2008). Study on the interaction between silver nanoparticles and nucleic acids in the presence of cetyltrimethylammonium bromide and its analytical application. Talanta.

[147] Adeyemi OS, Murata Y, Sugi T, Kato K. (2017). Inorganic nanoparticles kill Toxoplasma gondii via changes in redox status and mitochondrial membrane potential. Intl J Nanomed.

[148] Ullah I, Cosar G, Abamor ES, Bagirova M, Shinwari ZK, Allahverdiyev AM. (2018). Comparative study on the antileishmanial activities of chemically and biologically synthesized silver nanoparticles (AgNPs). 3 Biotech.

[149] Jalil PJ, Shnawa BH, Hamad SM. (2021). Silver nanoparticles: green synthesis, characterization, blood compatibility and protoscolicidal efficacy against echinococcus granulosus. Pak Vet J.

[150] Allahverdiyev AM, Abamor ES, Bagirova M, Ustundag CB, Kaya C, Kaya F (2011). Antileishmanial effect of silver nanoparticles and their enhanced antiparasitic activity under ultraviolet light. Int J Nanomed 2011.

[151] Choi O, Hu Z. (2008). Size dependent and reactive oxygen species related nanosilver toxicity to nitrifying bacteria. Environ Sci Technol.

[152] Chang Y-N, Zhang M, Xia L, Zhang J, Xing G. (2012). The toxic effects and mechanisms of CuO and ZnO nanoparticles. Materials.

[153] Stohs SJ, Bagchi D. (1995). Oxidative mechanisms in the toxicity of metal ions. Free Radical Biol Med.

[154] Kim J-H, Cho H, Ryu S-E, Choi M-U. (2000). Effects of metal ions on the activity of protein tyrosine phosphatase VHR: highly potent and reversible oxidative inactivation by Cu^2+^ ion. Arch Biochem Biophys.

[155] Saad AHA, Soliman MI, Azzam AM, Mostafa AB. (2015). Antiparasitic activity of silver and copper oxide nanoparticles against *Entamoeba histolytica* and *Cryptosporidium parvum* cysts. J Egypt Soc Parasitol.

[156] Zheng Y, Hou L, Liu M, Newell SE, Yin G, Yu C (2017). Effects of silver nanoparticles on nitrification and associated nitrous oxide production in aquatic environments. Sci Adv.

[157] Fabrega J, Luoma SN, Tyler CR, Galloway TS, Lead JR. (2011). Silver nanoparticles: behaviour and effects in the aquatic environment. Environ Int.

[158] Farghali M, Andriamanohiarisoamanana FJ, Ahmed MM, Kotb S, Yamashiro T, Iwasaki M (2019). Impacts of iron oxide and titanium dioxide nanoparticles on biogas production: Hydrogen sulfide mitigation, process stability, and prospective challenges. J Environ Manag.

[159] Kanhed P, Birla S, Gaikwad S, Gade A, Seabra AB, Rubilar O (2014). *In vitro* antifungal efficacy of copper nanoparticles against selected crop pathogenic fungi. Materials Lett.

[160] Mahmoodi S, Elmi A, Hallaj-Nezhadi S. (2018). Copper nanoparticles as antibacterial agents. J Mol Pharm Org Process Res.

[161] Al-Hakkani MF. (2020). Biogenic copper nanoparticles and their applications: a review. SN Appl Sci.

[162] Malekifard F, Tavassoli M, Vaziri K. (2020). *In vitro* assessment antiparasitic effect of selenium and copper nanoparticles on Giardia deodenalis cyst. Iran J Parasitol.

[163] Chatterjee AK, Chakraborty R, Basu T. (2014). Mechanism of antibacterial activity of copper nanoparticles. Nanotechnology.

[164] Elmore S. (2007). Apoptosis: A review of programmed cell death. Toxicol Pathol.

[165] Ezzatkhah F, Khalaf AK, Mahmoudvand H. (2021). Copper nanoparticles: Biosynthesis, characterization, and protoscolicidal effects alone and combined with albendazole against hydatid cyst protoscoleces. Biomed Pharmacother.

[166] Zhao Y, Jiang X. (2013). Multiple strategies to activate gold nanoparticles as antibiotics. Nanoscale.

[167] Sereemaspun A, Rojanathanes R, Wiwanitkit V. (2008). Effect of gold nanoparticle on renal cell: an implication for exposure risk. Renal Failure.

[168] Wiwanitkit V, Sereemaspun A, Rojanathanes R. (2009). Effect of gold nanoparticle on the microscopic morphology of white blood cell. Cytopathology.

[169] Shrivastava AK, Kumar S, Smith WA, Sahu PS. (2017). Revisiting the global problem of cryptosporidiosis and recommendations. Trop Parasitol.

[170] Yan D, Li Y, Liu Y, Li N, Zhang X, Yan C. (2021). Antimicrobial properties of chitosan and chitosan derivatives in the treatment of enteric infections. Molecules.

[171] Zahedi A, Paparini A, Jian F, Robertson I, Ryan U. (2016). Public health significance of zoonotic Cryptosporidium species in wildlife: critical insights into better drinking water management. Int J Parasitol Parasites Wildl.

[172] Tiwari G, Tiwari R, Sriwastawa B, Bhati L, Pandey S, Pandey P (2012). Drug delivery systems: an updated review. Int J Pharm Invest.

[173] Gerace E, Presti VDML, Biondo C. (2019). Cryptosporidium infection: epidemiology, pathogenesis, and differential diagnosis. Eur J Microbiol Immunol.

